# Ultrasound–Clinical Machine Learning Models for Differentiating Early Cervical Cancer from Myoma: A Retrospective Exploratory Study

**DOI:** 10.3390/jcm15093300

**Published:** 2026-04-26

**Authors:** Li Yin, Fajin Lv

**Affiliations:** State Key Laboratory of Ultrasound in Medicine and Engineering, Chongqing Medical University, Chongqing 400016, China; yl13935668816@163.com

**Keywords:** ultrasonic diagnosis, cervical cancer, cervical myoma, machine learning, magnetic resonance imaging

## Abstract

**Objective:** To develop machine learning models by integrating transvaginal ultrasound (TVUS) with clinical indicators, conduct visual analysis of the models, and systematically assess their diagnostic efficacy in differentiating early cervical neoplastic lesions. **Methods:** A total of 144 eligible patients (84 cases of early cervical cancer and 60 cases of cervical myoma) admitted to the First Affiliated Hospital of Chongqing Medical University from January 2018 to August 2025 were retrospectively enrolled in this study. Their clinical data, human papillomavirus (HPV) test results, Thinprep Cytologic Test (TCT) findings, TVUS images and magnetic resonance (MR) imaging data were collected and subjected to comprehensive statistical analysis. Univariate and multivariate Logistic Regression analyses were performed to identify independent differentiating factors for lesion classification. Eleven machine learning models were subsequently constructed, and their diagnostic performance was evaluated using receiver operating characteristic (ROC) curves, decision curve analysis (DCA), and the DeLong test. Finally, a nomogram was developed based on the optimal-performing model for clinical visualization. **Results:** The TVUS–clinical indicator integration model identified five independent differentiating factors: HPV status, TCT findings, menopausal status, ultrasonic tumor blood supply, and ultrasonic tumor morphology. In contrast, the MR–clinical indicator integration model screened out three independent factors: HPV status, TCT findings, and intratumoral signal intensity on MR T2-weighted imaging (T2WI). The TVUS integration model demonstrated marginally superior diagnostic performance, with a sensitivity of 0.988, specificity of 0.983, and an area under the ROC curve (AUC) of 0.991, compared with the MR integration model (sensitivity: 0.952, specificity: 0.950, AUC: 0.975); however, this difference in AUC values was not statistically significant (*p* = 0.911). Among the 11 machine learning models, the Logistic Regression model exhibited optimal classification performance and stability. DCA curves confirmed that all constructed models outperformed single-index diagnostic strategies in clinical decision-making for lesion differentiation. A nomogram was further established based on the Logistic Regression model for intuitive clinical application. **Conclusions:** Multiple machine learning models integrating TVUS with clinical indicators are successfully developed, and a corresponding nomogram is constructed in this study.

## 1. Introduction

Cervical cancer is the most prevalent malignant tumor of the female reproductive system, ranking eighth in global cancer incidence and far exceeding other gynecologic malignancies such as ovarian and endometrial cancer. Human papillomavirus (HPV) infection is well established as the primary etiological factor of cervical cancer [1]. In 2022, there were approximately 661,000 new cases and 348,000 cancer-related deaths worldwide, posing a severe threat to women’s health and life expectancy [1]. Cervical myoma is a common benign neoplastic lesion of the cervix, and it often presents with similar imaging features to early cervical cancer in initial clinical examinations [2]. As a special subtype of uterine myoma, cervical myoma shares overlapping clinical manifestations with early cervical cancer, including cervical enlargement, abnormal vaginal bleeding, and compressive symptoms [3].

A variety of clinical examinations are available for the evaluation of cervical neoplastic lesions, and the accuracy of differential diagnosis directly guides subsequent clinical decision-making. According to previous studies, ultrasonic imaging is the first-line modality for the initial screening of cervical neoplastic lesions [4]. As a routine gynecological imaging modality, transvaginal ultrasound (TVUS) is one of the methods for the initial detection and differentiation of cervical cancer and uterine myoma [5,6,7]. HPV testing and Thinprep Cytologic Test (TCT) are also validated as effective initial screening approaches for cervical malignancy, which can significantly reduce the mortality of early cervical cancer and are currently recognized as the gold-standard screening tools for early cervical cancer worldwide [8,9]. In comparison with TVUS, magnetic resonance imaging (MRI) has been widely used for the precise assessment of tumor infiltration range and distant metastasis due to its high soft tissue resolution, and it is also applied as an auxiliary modality for the differential diagnosis of cervical neoplastic lesions [10,11,12].

The therapeutic strategies for benign and malignant cervical neoplasms are fundamentally distinct, making the accuracy of initial diagnosis critical to the prognosis of clinical treatment. Although multiple clinical and imaging indicators are available for distinguishing benign and malignant cervical lesions, the weight of each indicator in clinical decision-making has not been thoroughly investigated. In recent years, artificial intelligence has advanced rapidly, with its applications in clinical practice expanding increasingly. Machine learning enables the effective quantification of complex clinical indicators and provides valuable support for solving various clinical decision-making problems. However, there remain few reports on the application of integrated clinical and imaging indicators in machine learning models for the differential diagnosis of early cervical neoplastic lesions.

Therefore, this study aimed to develop machine learning models centered on TVUS and integrated with other clinical indicators, perform visual analysis of these models exploratorily for the differentiation of early cervical neoplastic lesions, and evaluate their diagnostic efficacy.

## 2. Materials and Methods

### 2.1. Study Subjects

**Figure 1:**
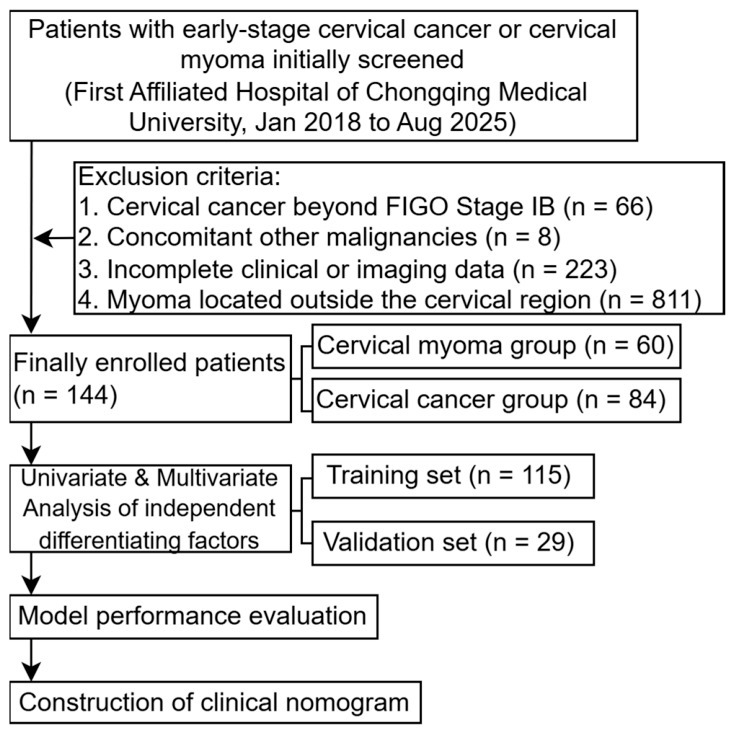
Flowchart.

A total of 1252 patients with early cervical cancer or cervical myoma admitted to the First Affiliated Hospital of Chongqing Medical University from January 2018 to August 2025 were initially screened for this study (Figure 1).

Inclusion criteria are as follows: (1) patients with pathologically confirmed early cervical cancer (FIGO Stage IA and IB); (2) patients with complete clinical data, valid HPV and TCT results, and complete TVUS and MRI data, with all examinations completed prior to surgery; and (3) patients with pathologically confirmed uterine myoma located in the cervical region. Exclusion criteria are as follows: (1) patients with early cervical cancer beyond FIGO Stage IB (only patients with tumor tissue confined to the cervix were included); (2) patients with other concurrent malignant tumors; (3) patients with incomplete clinical or imaging data; and (4) patients with uterine myoma located outside the cervical region.

Clinical and gynecological examination data, as well as imaging data of the eligible patients, were collected, including age, body mass index (BMI), gravidity and parity, menarche age, menstrual duration, menopausal status, clinical reports of HPV and TCTs, and formal diagnostic reports and raw images of TVUS and MR imaging. A total of 144 patients were finally enrolled in the study after strict screening based on the above criteria.

This study was approved by the Institutional Review Board of the First Affiliated Hospital of Chongqing Medical University, and the requirement for informed consent from patients was waived (Approval No.: KX2025-KYC0517-01). All research procedures were conducted in accordance with the Declaration of Helsinki.

### 2.2. Instruments and Examination Protocols

TVUS examinations were performed using GE Voluson E8 (78 examinations), GE Healthcare, Waukesha, WI, USA, and Toshiba Aplio 300 (66 examinations) ultrasound diagnostic scanners, Canon Medical Systems Corporation, Tochigi, Japan, equipped with transvaginal probes RIC6-12-D and PVT-712 BT (frequency range: 4–13 MHz), respectively. The standardized examination protocol was as follows: patients were required to empty their bladders, lie supine on the examination table in the lithotomy position, and the transvaginal probe was gently inserted into the vagina to make direct contact with the cervix for scanning.

MR imaging was performed using a GE 3.0T Signa HDxt MRI scanner, GE Healthcare, Waukesha, WI, USA, with an 8-channel abdominal coil. Standard axial and sagittal scans were acquired, including T1-weighted imaging (T1WI), T2-weighted imaging (T2WI), and contrast-enhanced T1WI sequences.

Two attending radiologists with more than 10 years of clinical experience in gynecological imaging independently analyzed the images of all patients. The inter-observer agreement kappa values ranged from 0.84 to 0.92, and the intra-observer agreement kappa values ranged from 0.88 to 0.95, both indicating good consistency. For TVUS images, the observers evaluated the mass echo intensity, boundary morphology, and blood supply characteristics. For MR images, the signal intensity, signal homogeneity, boundary between the mass and cervical myometrium, and degree of signal enhancement in T1WI, T2WI, and contrast-enhanced sequences were assessed systematically.

### 2.3. Construction of Machine Learning Models

Eleven general classifiers were selected to construct machine learning models for lesion differentiation, including Support Vector Machine (SVM), K-Nearest Neighbors (KNN), Random Forest, Extra Trees, eXtreme Gradient Boosting (XGBoost), Light Gradient Boosting Machine (LightGBM), Naive Bayes, Adaptive Boosting (AdaBoost), Gradient Boosting Machine, Logistic Regression (LR), and Multi-Layer Perceptron (MLP). The input variables of the models were the independent diagnostic factors identified from the integration of TVUS and clinical indicators. Finally, the model with the optimal diagnostic efficacy and stability was selected to develop a clinical nomogram for visual diagnosis.

### 2.4. Statistical Analysis

SPSS 27.0, IBM, Armonk, NY, USA and Python 3.11.5 software, Python Software Foundation, Wilmington, DE, USA, were used for statistical analysis, chart plotting, and machine learning model construction and evaluation. The Kolmogorov–Smirnov test and Levene test were applied to assess the normality of data distribution and homogeneity of variance, respectively. Normally distributed continuous data were expressed as mean ± standard deviation, while skewed distributed continuous data were presented as median (interquartile range). The independent samples *t*-test was used for the comparison of normally distributed continuous variables, and the Mann–Whitney U test was applied for skewed distributed continuous variables. The Pearson chi-square test was used for the comparison of categorical variables. Stepwise Logistic Regression analysis was performed for multivariate analysis to screen out independent predictive factors for early cervical neoplastic lesions. ROC curves were plotted to evaluate the diagnostic efficacy of the models, and the AUC value and other diagnostic parameters were calculated. The DeLong test was used for pairwise comparison of AUC values between different models. For correlation analysis, the Spearman rank correlation coefficient was calculated: an absolute correlation coefficient < 0.1 indicated no correlation, 0.1–0.5 indicated a moderate correlation, and >0.5 indicated a strong correlation. A two-tailed *p* < 0.05 was considered statistically significant for all analyses.

## 3. Results

### 3.1. Clinical and Imaging Characteristics of the Study Population

**Figure 2:**
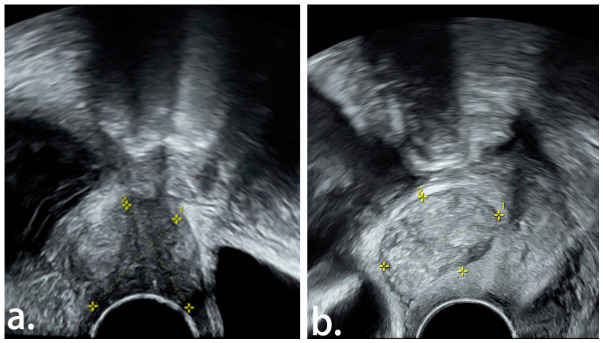
Comparative abdominal TVUS images of cervical myoma and early cervical cancer. (**a**) A moderate-echo solid tumor (2.79 cm × 2.77 cm) was detected in the cervix, presenting with irregular morphology, partially ill-defined borders, and heterogeneous echo; pathologically confirmed as FIGO Stage IB2 cervical cancer after surgery. (**b**) A moderate-echo tumor (3.07 cm × 2.06 cm) was identified in the cervix, with well-defined borders and shallow lobulation; pathologically confirmed as a cervical myoma after surgery. Yellow cross calipers, numbered labels, and dashed lines in the figure denote the ultrasound measurement markers, which are used to measure the tumor’s maximum diameter (1) and anteroposterior diameter (2) and delineate the lesion boundaries.

A total of 144 patients with early cervical cancer or cervical myoma who met the inclusion and exclusion criteria were enrolled in the study, including 84 cases of early cervical cancer and 60 cases of cervical myoma. The baseline characteristics of the study population were as follows: mean age was 45.57 ± 9.84 years, mean BMI was 23.32 ± 3.21 kg/m^2^, and 36 patients (25.0%) were postmenopausal. The typical TVUS manifestations of early cervical cancer and cervical myoma are shown in Figure 2, and the comparative analysis of clinical, TVUS, and MR imaging characteristics between the two groups is presented in Table 1.

### 3.2. Identification of Independent Differentiating Factors

To construct the TVUS–clinical indicator integration model and MR–clinical indicator integration model, the pathological diagnosis (cervical myoma vs. early cervical cancer) was set as the dependent variable, and the clinical and imaging indicators with statistically significant differences in univariate analysis were selected as independent variables. Collinearity diagnosis was performed for the included variables, and all variables had a variance inflation factor (VIF) < 5, indicating no severe multicollinearity. Binary Logistic Regression analysis was then performed to establish the two diagnostic models and screen out independent differentiating factors.

The results showed that the TVUS–clinical indicator integration model identified five independent factors: HPV status (*p* = 0.005), TCT findings (*p* < 0.001), menopausal status (*p* = 0.022), ultrasonic tumor blood supply (CDFI) (*p* = 0.019), and ultrasonic tumor morphology (*p* = 0.039). The MR–clinical indicator integration model screened out three independent factors: HPV status (*p* = 0.005), TCT findings (*p* < 0.001), and intratumoral signal intensity on MR T2WI (*p* = 0.005). The detailed results of the Logistic Regression analysis are presented in Table 2.

### 3.3. Comparative Analysis of Diagnostic Efficacy of Different Models

**Figure 3:**
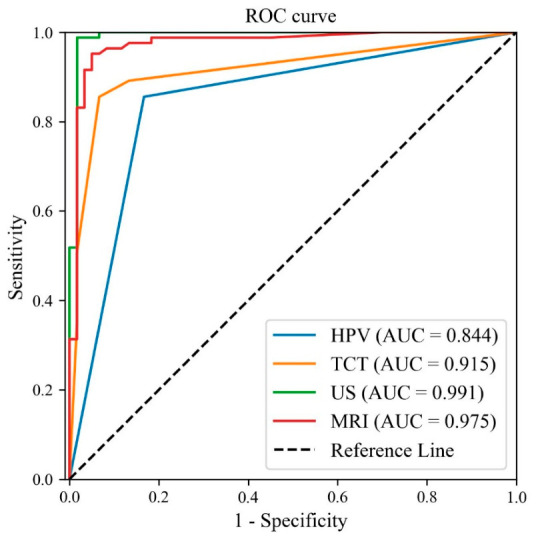
ROC curves of the TVUS-clinical, MR–clinical indicator integration models, and single indicators. Note: The green line represents the TVUS–clinical indicator integration model, the red line represents the MR–clinical indicator integration model, the orange line represents the single TCT indicator, and the blue line represents the single HPV indicator. The dashed line is the reference line. The horizontal axis is 1-specificity, and the vertical axis is sensitivity. HPV (AUC = 0.844), TCT (AUC = 0.915), TVUS (AUC = 0.991), and MRI (AUC = 0.975).

Logistic Regression models were constructed based on the five independent factors of the TVUS–clinical indicator integration model and the three independent factors of the MR–clinical indicator integration model, respectively (Figure 3). The DeLong test was then used for pairwise comparison of the AUC values to evaluate the diagnostic efficacy of the models.

The results showed that the TVUS–clinical indicator integration model had a sensitivity of 0.988, specificity of 0.983, and overall accuracy of 0.986; the MR–clinical indicator integration model had a sensitivity of 0.952, specificity of 0.950, and overall accuracy of 0.951. The AUC values of the TVUS–clinical indicator integration model, MR–clinical indicator integration model, single TCT indicator, and single HPV indicator were 0.991, 0.975, 0.915, and 0.844, respectively, with the TVUS integration model showing the highest AUC value. However, the DeLong test confirmed that there was no statistically significant difference in AUC values among the four models (all *p* > 0.05).

### 3.4. Correlation Analysis of TVUS, MR Features and Clinical Indicators

**Figure 4:**
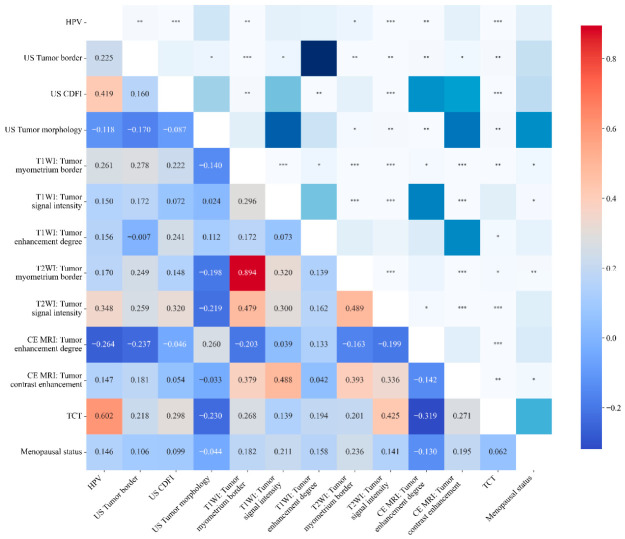
Spearman correlation heatmap of clinical and imaging indicators. Note: The color gradient reflects the Spearman correlation coefficient, with red indicating a positive correlation and blue indicating a negative correlation. Asterisks in the upper part represent statistical significance: * *p* < 0.05; ** *p* < 0.01; *** *p* < 0.001. The numerical values in the lower part are the Spearman correlation coefficients.

To explore the interrelationship between various clinical and imaging indicators, the Spearman rank correlation test was performed to analyze the correlation between TVUS features, MR features, HPV status, TCT findings, and other clinical indicators (Figure 4). The results showed that ultrasonic tumor blood supply was positively correlated with HPV status (r = 0.419, *p* < 0.001) and TCT findings (r = 0.298, *p* < 0.01). The ultrasonic tumor–myometrium border was also positively correlated with HPV status (r = 0.225, *p* < 0.01) and TCT findings (r = 0.298, *p* < 0.01).

### 3.5. Construction and Evaluation of Machine Learning Models

**Figure 5:**
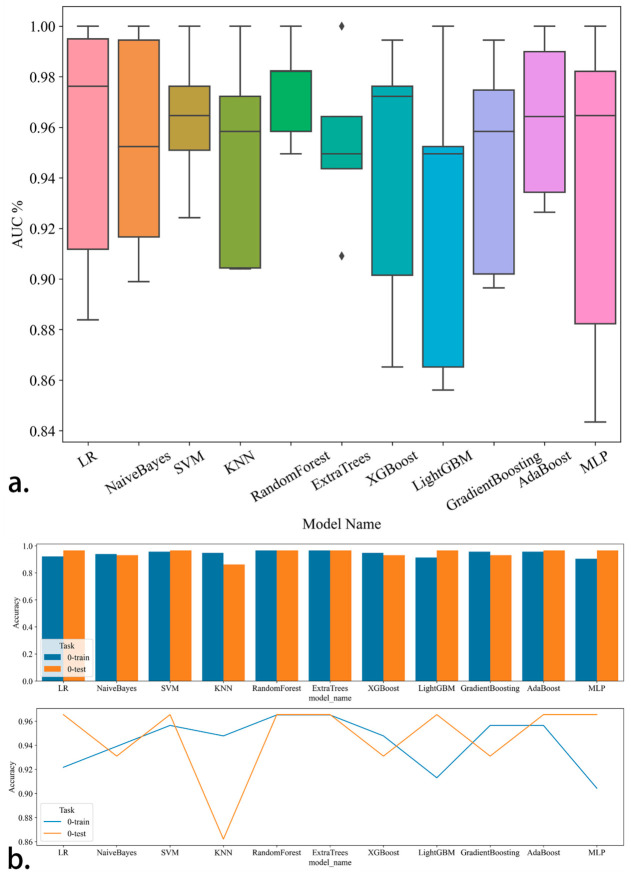
Comparative analysis of the diagnostic efficacy of different machine learning models. Note: (**a**). Distribution characteristics of binary classification performance: box plot of AUC values of different models. Horizontal axis: model name; vertical axis: AUC percentage. Each box represents the interquartile range of AUC values; The diamond symbols in the box plot represent outliers (extreme values) in the distribution of AUC values for each machine learning model. (**b**). Generalization ability comparison: the upper part is a bar chart of the accuracy of each model in the training set and validation set; the lower part is a line chart of the accuracy trend. Horizontal axis: model name; vertical axis: accuracy (the proportion of correctly classified samples).

**Figure 6:**
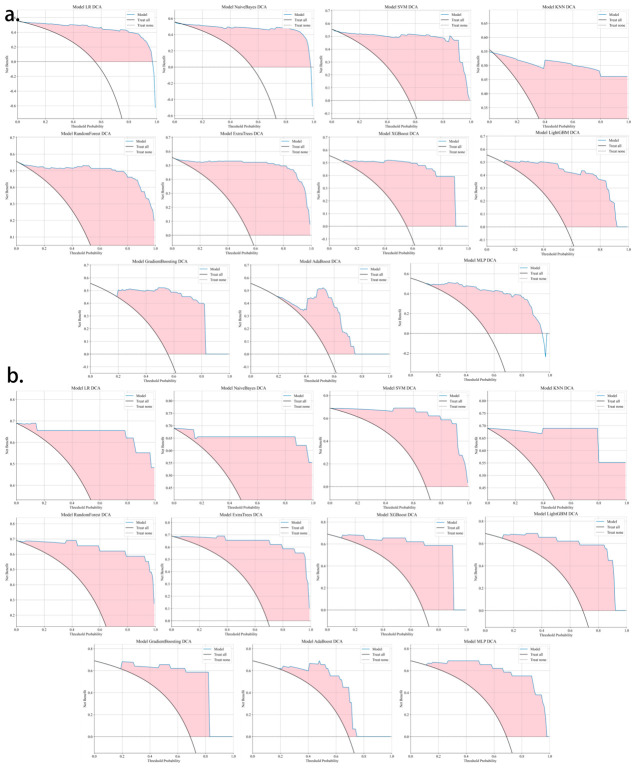
DCA curves of different machine learning models in the training set and validation set. Note: Decision curve analysis (DCA). Horizontal axis (X−axis): Threshold probability. Vertical axis (Y−axis): Net benefit. (**a**) (Upper three groups): DCA curve verification of the training set; (**b**) (lower three groups): DCA curve verification of the validation set.

**Figure 7:**
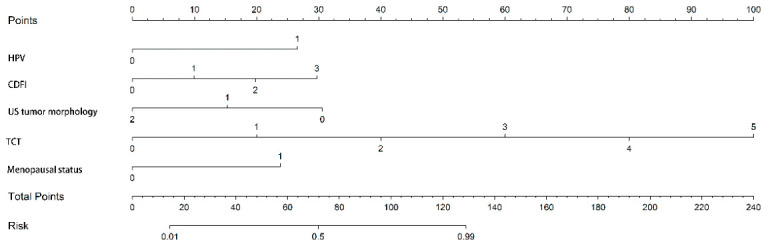
Nomogram of the Logistic Regression model for predicting early cervical malignant neoplasms. Note: The predictive factors (on the left) are the independent differentiating factors: HPV status, ultrasonic tumor blood supply, ultrasonic tumor morphology, TCT findings, and menopausal status. The top line is the score axis (0~100 points). The Total Points axis is the sum of the scores of the above factors, and the total score is vertically projected onto this axis. The bottom is the risk axis, and the total score is vertically projected onto this axis to obtain the predicted probability of malignant cervical neoplasms in an individual patient.

Eleven machine learning models were constructed based on the training set data (Figure 5). In terms of AUC performance and model stability, the Logistic Regression model exhibited the optimal classification performance among all 11 models, followed by the Naive Bayes model. The detailed performance of all models in the training set and validation set is presented in Table 3. The diagnostic efficacy of these machine learning models was further evaluated by DCA (Figure 6), and the results confirmed that all models could effectively screen out malignant cervical neoplasms in clinical decision-making and outperformed single-index diagnostic strategies. Finally, based on the Logistic Regression model, a nomogram was developed to visualize the clinical decision-making for early cervical neoplastic lesions, providing an accurate and non-invasive diagnostic tool for clinical practice (Figure 7).

## 4. Discussion

In this study, univariate analysis revealed that HPV status, TCT findings, menopausal status, TVUS tumor morphology and border, CDFI tumor blood flow signal, MR T1WI tumor border, signal intensity and intratumoral homogeneity, MR T2WI tumor border and signal intensity, as well as the degree and homogeneity of intratumoral enhancement on contrast-enhanced MR sequences, were all statistically different between the cervical cancer group and cervical myoma group (all *p* < 0.05). In contrast, no statistically significant differences were observed in age, height, BMI, menarche age, gravidity, parity, menstrual duration, menstrual cycle, dysmenorrhea, menstrual regularity, menstrual volume, as well as TVUS features including intracervical echo, cervical enlargement, tumor echo intensity and intratumoral echo homogeneity between the two groups (all *p* > 0.05).

Previous studies have reported that in the initial screening of cervical cancer by TVUS, malignant lesions typically present with abundant, disordered, strip-like or branched intratumoral color blood flow signals with low-resistance arterial spectra (RI < 0.40) on CDFI, while cervical myomas show relatively weak blood flow signals that are mostly peripherally distributed around the tumor with sparse internal flow [13]. Degenerated cervical myomas exhibit heterogeneous intratumoral echo, which makes them difficult to differentiate from early cervical cancer [13]. Consistent with these findings, the present study found that early cervical cancer also presented with abundant blood flow signals on CDFI, and no significant differences in intratumoral echo intensity and homogeneity were observed between early cervical cancer and cervical myoma. Cervical myomas typically show round or subround morphology on imaging due to their benign growth pattern, while early cervical cancer presents irregular or lobulated morphology, ill-defined borders and no complete capsule due to its invasive growth characteristics [14,15]. In this study, the TVUS images of early cervical cancer mostly showed irregular morphology, which was significantly different from that of cervical myoma and could serve as a key differentiating feature.

Early cervical cancer and cervical myoma have completely different prognoses and require distinct therapeutic strategies, which highlights the unique value of TVUS in the differential diagnosis of these two lesions. Previous studies have suggested that TVUS is less sensitive than MRI in the detection of early cervical cancer; however, neither TVUS nor MRI can effectively display early invasive foci of FIGO Stage IA cervical cancer on morphological images, which often leads to missed diagnosis of early lesions [11]. In the present study, the TVUS–clinical indicator integration model demonstrated comparable diagnostic efficacy to the MR–clinical indicator integration model in the diagnosis of early cervical cancer, with a marginal superiority over the latter. These findings confirm that TVUS has higher diagnostic value for the differentiation of benign and malignant early cervical neoplastic lesions when integrated with clinical indicators.

HPV testing and TCT have been clinically validated as the gold-standard screening tools for early cervical cancer, and they play an irreplaceable role in the detection of precancerous lesions and early invasive cancer [9]. However, recent reports have indicated that although TCT has high specificity, its false negative rate is relatively high due to variations in clinicians’ operating techniques and the location of lesions in early cytological examinations. In addition, both vaginal and cervical diseases can lead to positive results for high-risk HPV, resulting in a high false positive rate of HPV testing [8]. Therefore, screening for early cervical cancer using only HPV testing and TCT is prone to misdiagnosis and missed diagnosis. Moreover, previous studies have shown that cervical malignant neoplasms are more likely to occur in postmenopausal women, which is distinct from cervical myomas that mainly affect reproductive-age women due to hormonal regulation [2]. In the present study, menopausal status was identified as an independent diagnostic factor, which also plays a valuable role in the differential diagnosis of cervical neoplastic lesions. A comparative analysis of diagnostic efficacy confirmed that the TVUS–clinical indicator integration model outperformed the MR–clinical indicator integration model, which validates the effectiveness of early TVUS screening integrated with clinical indicators for the differential diagnosis of cervical neoplastic lesions.

Based on the independent diagnostic factors identified from the TVUS–clinical indicator integration, 11 machine learning models were constructed and validated in this study. All models exhibited good diagnostic efficacy and could effectively differentiate benign and malignant cervical neoplasms, with the Logistic Regression model showing the optimal performance in all aspects. Finally, the clinical independent differentiating factors were visualized based on the Logistic Regression classifier, which intuitively reflects the internal decision-making process of the classifier and the weight of each indicator in the differential diagnosis. Therefore, the integration of TVUS with clinical models forms a complementary diagnostic strategy.

This study has several limitations that should be acknowledged. First, it is a retrospective single-center study with a relatively small sample size, and the lack of external validation is an inherent limitation of this preliminary work. Future studies should expand the sample size to improve the robustness of the models. Second, only qualitative TVUS features were included in the present study; the introduction of quantitative TVUS indicators, such as the resistance index (RI), in future studies can further quantify the hemodynamic characteristics of cervical lesions and improve the objectivity and accuracy of differential diagnosis.

## 5. Conclusions

Multiple machine learning models integrating TVUS with clinical indicators are successfully developed in this study, and a nomogram is constructed to visualize the decision weight of the classifier.

## Figures and Tables

**Table 1:** Comparison of clinical data, TVUS and MR imaging features between patients with cervical myoma and early cervical cancer.

Variables	Cervical Myoma Group (n = 60)	Cervical Cancer Group (n = 84)	*p* Value
Clinical Indicators			
Age (Years) ^a^	43.58 ± 8.85	46.99 ± 10.26	0.081
Body Weight (kg) ^b^	56.5 (52.0–60.5)	57.75 (52.75–62.0)	0.724
Height (cm) ^a^	157.6 ± 5.08	156.71 ± 5.13	0.492
BMI (kg/m^2^) ^a^	22.94 ± 2.76	23.6 ± 3.47	0.168
Menarche Age (Years) ^b^	13.0 (13.0–14.0)	13.5 (12.0–15.0)	0.747
Gravidity ^b^	2.0 (2.0–4.0)	3.0 (2.0–4.0)	0.177
Parity ^b^	1.0 (1.0–2.0)	1.0 (1.0–2.0)	0.146
Menstrual Duration (Days) ^b^	5.5 (5.0–6.25)	5.0 (4.0–7.0)	0.698
Menstrual Cycle (Days) ^b^	29.0 (26.0–30.0)	28.0 (26.75–30.0)	0.236
Dysmenorrhea (No/Yes) ^c^	50/10 (83.3/16.7)	76/8 (90.5/9.5)	0.307
Menstrual Regularity (Regular/Irregular) ^c^	46/14 (76.7/23.3)	56/28 (66.7/33.3)	0.265
Past Menstrual Volume (Normal/Low/High) ^c^	36/6/18 (60.0/10.0/30.0)	52/8/24 (61.9/9.5/28.6)	0.974
HPV (Negative/Positive) ^c^	50/10 (83.3/16.7)	12/72 (14.3/85.7)	<0.001
TCT (Normal/Inflammation/ASCUS/AGC/LSIL/ASC-H/HSIL) ^c^	52/4/3/0/0/1 (86.7/6.7/5.0/0/0/1.7)	9/3/29/9/4/30 (10.7/3.6/34.5/10.7/4.8/35.7)	<0.001
Menopausal Status (Premenopausal/Postmenopausal) ^c^	51/9 (85.0/15.0)	57/27 (67.9/32.1)	0.032
TVUS indicators			
Intracervical Echo (Homogeneous/Heterogeneous) ^c^	4/56 (6.7/93.3)	3/81 (3.6/96.4)	0.647
Cervical Enlargement (No/Yes) ^c^	24/36 (40.0/60.0)	37/47 (44.0/56.0)	0.754
Tumor Echo Intensity (Low/Moderate/High) ^c^	42/12/6 (70.0/20.0/10.0)	52/27/5 (61.9/32.1/6.0)	0.222
Intratumoral Echo Homogeneity (Homogeneous/Heterogeneous) ^c^	8/52 (13.3/86.7)	9/75 (10.7/89.3)	0.827
Tumor Morphology (Round/Regular/Irregular) ^c^	17/13/30 (28.3/21.7/50.0)	1/22/61 (1.2/26.2/72.6)	<0.001
Tumor Border (Clear/Ill-defined) ^c^	10/50 (16.7/83.3)	1/83 (1.2/98.8)	0.002
CDFI Tumor Blood Flow Signal (Absent/Minimal/Moderate/Abundant) ^c^	5/11/39/5 (8.3/18.3/65.0/8.3)	46/14/14/10 (54.8/16.7/16.7/11.9)	<0.001
MRI Indicators			
T1WI Sequence			
Tumor–Myometrium Border (Clear/Ill-defined/Obscure) ^c^	44/12/4 (73.3/20.0/6.7)	26/22/36 (31.0/26.2/42.9)	<0.001
Intratumoral Signal Homogeneity (Homogeneous/Heterogeneous) ^c^	33/27 (55.0/45.0)	29/55 (34.5/65.5)	0.011
Intratumoral Signal Intensity (Low/Moderate/High) ^c^	14/46/0 (23.3/76.7/0)	3/78/3 (3.6/92.9/3.6)	<0.001
T2WI Sequence			
Tumor–Myometrium Border (Clear/Ill-defined/Obscure) ^c^	40/11/9 (66.7/18.3/15.0)	30/11/43 (35.7/13.1/51.2)	<0.001
Intratumoral Signal Homogeneity (Homogeneous/Heterogeneous) ^c^	22/38 (36.7/63.3)	28/56 (33.3/66.7)	0.813
Intratumoral Signal Intensity (Low/Moderate/High) ^c^	25/27/8 (41.7/45.0/13.3)	6/16/62 (7.1/19.0/73.8)	<0.001
Contrast-Enhanced Sequence			
Intratumoral Enhancement Degree (Low/Moderate/High) ^c^	13/7/40 (21.7/11.7/66.7)	27/36/21 (32.1/42.9/25.0)	<0.001
Intratumoral Enhancement Homogeneity (Homogeneous/Heterogeneous) ^c^	35/25 (58.3/41.7)	25/59 (29.8/70.2)	0.001

Note: TVUS = transvaginal ultrasound; CDFI = color Doppler flow imaging; HPV = human papillomavirus; TCT = Thinprep Cytological Test; ASCUSs = atypical squamous cells of undetermined significance; AGCs = atypical glandular cells; LSIL = low-grade squamous intraepithelial lesion; ASC-Hs = atypical squamous cells—cannot exclude HSIL; and HSIL = high-grade squamous intraepithelial lesion; MRI = magnetic resonance imaging. Continuous variables conforming to normal distribution were expressed as mean ± standard deviation (x ± s), and those with non-normal distribution were expressed as median (interquartile range, M (P25–P75)). Categorical variables were presented as a case number (percentage): ^a^ independent-sample *t*-test; ^b^ Mann–Whitney U test; and ^c^ Pearson chi-square test.

**Table 2:** Logistic Regression analysis of independent factors for differentiating early cervical cancer and cervical myoma.

Independent Factors	B	Standard Error	Wald χ^2^	df	Exp(B)	*p* Value	95% CI
TVUS–clinical indicator integration model							
HPV	2.006	0.721	7.733	1	7.430	0.005	1.808–30.543
TCT	1.696	0.387	19.197	1	5.453	<0.001	2.553–11.646
Menopausal status	1.936	0.847	5.228	1	6.932	0.022	1.319–36.438
Tumor–myometrium border (TVUS)	2.186	1.509	2.096	1	8.895	0.148	0.046–17.14
Tumor blood supply (CDFI)	0.833	0.354	5.525	1	2.299	0.019	1.148–4.604
TVUS tumor morphology	−1.270	0.614	4.275	1	0.281	0.039	0.084–0.936
Constant	−5.378	1.709	9.899	1	0.005	0.002	0.18–3.05
MR–clinical indicator integration model							
HPV	4.071	1.326	9.418	1	58.600	0.002	0.043–7.89
TCT	1.675	0.544	9.479	1	5.338	0.002	1.838–15.503
Menopausal status	2.060	1.278	2.601	1	7.850	0.107	0.064–9.60
T1WI tumor–myometrium border	2.204	1.334	2.729	1	9.060	0.099	0.07–12.38
T1WI intratumoral signal homogeneity	1.363	1.430	0.908	1	3.906	0.341	0.02–6.44
T1WI intratumoral signal intensity	4.270	3.799	1.263	1	71.555	0.261	0.00–12.26
T2WI tumor–myometrium border	−1.349	1.121	1.448	1	0.259	0.229	0.029–2.336
T2WI intratumoral signal intensity	2.804	0.994	7.962	1	16.510	0.005	0.23–11.58
Contrast-enhanced intratumoral enhancement degree	−0.106	0.643	0.027	1	0.899	0.869	
Contrast-enhanced intratumoral enhancement homogeneity	−0.348	1.244	0.078	1	0.706	0.780	

Note: TVUS = transvaginal ultrasound; CDFI = color Doppler flow imaging; HPV = human papillomavirus; TCT = Thinprep Cytological Test; MR = magnetic resonance.

**Table 3:** Performance of 11 machine learning models in the training set and validation set.

Models	Training Set						Validation Set			
	AUC	95% CI	Sensitivity	Specificity	Accuracy	AUC	95% CI	Sensitivity	Specificity	Accuracy
SVM	0.981	0.9528–1.0000	0.937	0.980	0.957	1.000	1.0000–1.0000	0.950	1.000	0.966
KNN	0.983	0.9629–1.0000	0.812	1.000	0.896	1.000	1.0000–1.0000	0.950	1.000	0.966
RF	0.995	0.9864–1.0000	0.937	1.000	0.965	1.000	1.0000–1.0000	0.950	1.000	0.966
Extra Trees	0.997	0.9909–1.0000	0.000	1.000	0.443	1.000	1.0000–1.0000	0.950	1.000	0.966
XGBoost	0.985	0.9634–1.0000	0.937	0.980	0.957	0.994	0.9790–1.0000	0.900	1.000	0.931
Light GBM	0.945	0.8989–0.9902	0.844	0.922	0.878	1.000	1.0000–1.0000	0.950	1.000	0.966
Naive Bayes	0.965	0.9268–1.0000	0.906	0.980	0.939	0.994	0.9790–1.0000	0.900	1.000	0.931
AdaBoost	0.985	0.9658–1.0000	0.922	1.000	0.957	1.000	1.0000–1.0000	0.950	1.000	0.966
GBM	0.985	0.9634–1.0000	0.922	1.000	0.957	0.994	0.9790–1.0000	0.900	1.000	0.931
LR	0.965	0.9266–1.0000	0.953	0.922	0.939	1.000	1.0000–1.0000	0.950	1.000	0.966
MLP	0.950	0.9033–0.9971	0.969	0.824	0.904	1.000	1.0000–1.0000	0.950	1.000	0.966

Note: SVM: Support Vector Machine, KNN: K-Nearest Neighbors, RF: Random Forest, XGBoost: eXtreme Gradient Boosting, Light GBM: Light Gradient Boosting Machine, AdaBoost: Adaptive Boosting, Gradient Boosting Machine, GBM: Gradient Boosting Machine, LR: Logistic Regression, MLP: Multi-Layer Perceptron.

## Data Availability

The data that support the findings of this study are available from the corresponding author upon reasonable request.

## Abbreviations

HPV: Human Papillomavirus; TCT: Thinprep Cytologic Test; TVUS: Transvaginal Ultrasound; CDFI: Color Doppler Flow Imaging; MRI: Magnetic Resonance Imaging; T1WI: T1-Weighted Imaging; T2WI: T2-Weighted Imaging; IRB: Institutional Review Board.
